# Pigs as Source for Toxigenic *Corynebacterium ulcerans*

**DOI:** 10.3201/eid1508.081568

**Published:** 2009-08

**Authors:** Regina Schuhegger, Christoph Schoerner, Julia Dlugaiczyk, Ina Lichtenfeld, Alexander Trouillier, Veronique Zeller-Peronnet, Ulrich Busch, Anja Berger, Rudolf Kugler, Stefan Hörmansdorfer, Andreas Sing

**Affiliations:** National Consiliary Laboratory for Diphtheria, Oberschleißheim, Germany (R. Schuhegger, A. Berger, R. Kugler, A. Sing); Bavarian Health and Food Safety Authority, Oberschleißheim, (R. Schuhegger, V. Zeller-Peronnet, U. Busch, A. Berger, R. Kugler, S. Hörmansdorfer, A. Sing); University Clinic of Erlangen, Erlangen, Germany (C. Schoerner); University Hospital Erlangen, Erlangen (J. Dlugaiczyk); Klinikum Coburg, Coburg, Germany (I. Lichtenfeld); and Landratsamt (District Office) Coburg, Coburg (A. Trouillier)

**Keywords:** Pigs, diphtheria, zoonoses, Corynebacterium ulcerans, bacteria, letter

**To the Editor:** Toxigenic *Corynebacterium ulcerans* may cause a zoonotic infection similar to diphtheria caused by *C. diphtheriae*. Previously, dairy cattle were considered to be the main reservoir for *C. ulcerans* (*1*), but recent publications suggest pet dogs and pet cats as carriers (cats often show bilateral nasal discharge) (*2*). We report a case of severe *C. ulcerans* diphtheria-like disease in a person who had had contact with pigs.

In December 2007, a previously healthy 56-year-old female farmer was admitted to the Ear, Nose and Throat Department of the University Hospital Erlangen with a 1-week history of sore throat and progressive dysphagia. She did not report fever and had not received prior treatment with antimicrobial drugs. She had thick, whitish pseudomembranes on her uvula, pharynx, and both tonsils. Endoscopic examination of her larynx and hypopharynx showed that both vocal cords were mobile and the mucosa was erythematous. Enlarged cervical lymph nodes were palpable on both sides of her neck. She had no signs of cranial nerve palsies. Her temperature was 36.5°C. Because of the extensive oropharyngeal pseudomembranes, diphtheria was suspected and diphtheria antitoxin (30,000 IU) was administered intramuscularly. The patient was isolated and received intravenous penicillin (5 million units 4×/day).

A pharyngeal swab obtained from below the whitish pseudomembranes grew toxigenic *C. ulcerans*. Species identification was achieved by biochemical differentiation (API Coryne code 0111326), *rpoB* sequencing (*3*), and MALDI-TOF analysis (Microflex LT and Biotyper 2.0 Software; Bruker Daltonics, Bremen, Germany). Toxigenicity of the strain, named KL126, was verified by using a *C. diphtheriae tox*–PCR (*4*–*6*), a *C. ulcerans tox*–specific PCR (*4*), and the Elek test as described previously (*4*,*5*). The *tox* sequence (GenBank accession no. FJ858272) differs from 2 other published *C. ulcerans tox* sequences (AB304279.1 and AY703827.1) at only 3 bp.

The patient recovered quickly, and the pseudomembranes vanished within 2 days. However, because an allergic rash had developed after her third day of treatment with penicillin, antimicrobial drug treatment was switched to intravenous erythromycin (500 mg 4×/d). When 1 day later the standardized antibiogram showed resistance to erythromycin, the patient received intravenous ceftriaxone (2 g 1×/d) for 12 days. Seven days after initiation of antimicrobial drug therapy, pharyngeal swabs were taken on 3 consecutive days. Because *C. ulcerans* no longer grew on culture, the patient was discharged from the hospital. However, 2 days later she was readmitted to hospital for severe polyneuropathy with neuralgia and weakness of both arms, acute difficulty swallowing, and hoarseness. Signs of cardiomyopathy, including sinus bradycardia and grade I atrioventricular block, were present. The patient recovered after symptomatic treatment and returned home after 2 weeks. According to her records, the patient had received a basic vaccination against diphtheria in 1960 and a booster in 1998.

The literature describes the classic animal sources for toxigenic *C. ulcerans* as dairy cattle with mastitis (*1*). Since 2005, toxigenic *C. ulcerans* carriage in companion animals, e.g., pet cats and dogs, has been reported (*2*). Two cases of transmission of a toxigenic *C. ulcerans* strain from pet dogs to their immunocompromised female owners have been documented in France (*7*,*8*). In 2008, toxigenic *C. ulcerans* in 2 dead killer whales from a Japanese zoo was reported (*9*).

To determine the source of our patient’s illness, an outbreak investigation involving her family and their farm animals was conducted. Their medium-sized pig-breeding farm was located in a remote rural village surrounded by woods; they raised ≈500 pigs in a nonindustrialized manner, and no piglets were purchased from outside the farm. Pharyngeal swabs of 3 family members, 19 pigs, and the farm dog were analyzed for *C. ulcerans*. Although all family members and the dog were negative for *C. ulcerans*, 1 of the 19 asymptomatic pigs harbored a toxigenic strain of *C. ulcerans*. Sequencing of *rpoB* and *tox* showed 100% homology between the human and the pig strains. Ribotyping (*10*) confirmed this result, suggesting the identity of both strains; the obtained ribotype is similar to the reported U1 ribotype profile found in humans and cats (*2*).

We report proven transmission of a toxigenic *C. ulcerans* strain between a livestock animal and a human, as well as harboring of toxigenic *C. ulcerans* in pigs. Introduction of *C. ulcerans* from wild animals seems unlikely because the barn doors were reportedly closed at all times. Because handling of *C. ulcerans*–infected pigs may lead to diphtheria-like illnesses, studies of toxigenic *C. ulcerans* carriage among pigs are needed. Similar to our case, diphtheria-like disease caused by an erythromycin- and clindamycin-resistant toxigenic *C. ulcerans* strain in a US patient has been recently (in 2008) reported (*1*). Because current recommendations based on *C. diphtheriae*–caused disease consider erythromycin as the second-line option for treatment or postexposure prophylaxis, these findings highlight the importance of antimicrobial-drug susceptibility testing of toxigenic *C. ulcerans* strains.
